# *Escherichia coli* Nissle 1917 inhibits biofilm formation and mitigates virulence in *Pseudomonas aeruginosa*

**DOI:** 10.3389/fmicb.2023.1108273

**Published:** 2023-03-08

**Authors:** Ahmad M. Aljohani, Cecile El-Chami, Muna Alhubail, Ruth G. Ledder, Catherine A. O’Neill, Andrew J. McBain

**Affiliations:** ^1^Division of Pharmacy and Optometry, School of Health Sciences, Faculty of Biology, Medicine and Health, The University of Manchester, Manchester, United Kingdom; ^2^Ministry of Education, Riyadh, Saudi Arabia; ^3^Division of Musculoskeletal and Dermatological Science, Faculty of Biology, Medicine and Health, School of Biological Science, The University of Manchester, Manchester, United Kingdom

**Keywords:** probiotic, *Escherichia coli* Nissle 1917, biofilm, *Pseudomonas aeruginosa*, *Galleria mellonella*, virulence mitigation

## Abstract

In the quest for mitigators of bacterial virulence, cell-free supernatants (CFS) from 25 human commensal and associated bacteria were tested for activity against *Pseudomonas aeruginosa*. Among these, *Escherichia coli* Nissle 1917 CFS significantly inhibited biofilm formation and dispersed extant pseudomonas biofilms without inhibiting planktonic bacterial growth. eDNA was reduced in biofilms following exposure to *E. coli* Nissle CFS, as visualized by confocal microscopy. *E. coli* Nissle CFS also showed a significant protective effect in a *Galleria mellonella*-based larval virulence assay when administrated 24 h before challenge with the *P. aeruginosa.* No inhibitory effects against *P. aeruginosa* were observed for other tested *E. coli* strains. According to proteomic analysis, *E. coli* Nissle CFS downregulated the expression of several *P. aeruginosa* proteins involved in motility (Flagellar secretion chaperone FliSB, B-type flagellin fliC, Type IV pilus assembly ATPase PilB), and quorum sensing (acyl-homoserine lactone synthase lasI and HTH-type quorum-sensing regulator rhlR), which are associated with biofilm formation. Physicochemical characterization of the putative antibiofilm compound(s) indicates the involvement of heat-labile proteinaceous factors of greater than 30 kDa molecular size.

## Introduction

Various alternative approaches to combat infection are receiving research attention including targeting bacterial virulence. Virulence-associated phenotypes in bacteria include biofilm formation and other processes involved in colonization, invasion, and persistence (Theuretzbacher and Piddock, 2019). Factors involved include adhesions, toxins, and specialized secretion systems (Finlay and Falkow, 1989). Several strategies have been evaluated to inhibit bacterial adhesion to tissues and the formation of biofilms (Hall-Stoodley et al., 2004). It has been reported that inhibiting iron sequestration in *Pseudomonas aeruginosa* using gallium-mediated quenching of pyoverdine siderophores reduced virulence and proliferation in a larval infection model (Ross-Gillespie et al., 2014). Toxin-neutralization (Wei et al., 2017), and agents that interfere with quorum-sensing systems are also under evaluation (Defoirdt, 2018).

Quorum sensing signals which in gram-negative bacteria, include N-acyl homoserine lactones (AHLs), and peptides in gram-positive bacteria play a significant role in biofilm formation and virulence (Hauser et al., 2016) and are therefore promising targets for novel therapeutics. The main drug principles against these signaling processes are either inhibitors of molecule-receptor interactions or inhibitors of the synthesis of the signaling molecules (Rampioni et al., 2014).

Bacteria may be a sustainable and diverse source of compounds with potential anti-virulence activity. An exopolysaccharide of *L. acidophilus* has been reported to significantly decrease biofilm formation by enterohemorrhagic *E. coli* (EHEC) and a range of other bacteria, including *Salmonella*, *Yersinia enterocolitica*, *Listeria monocytogenes* by targeting autoaggregation and attachment (Kim et al., 2009). Supernatants of *Lactobacillus* species, including *L. plantarum*, *L. reuteri*, *L. casei*, and *L. salivarius*, are reported to inhibit the formation of biofilms by the dental caries-associated bacterium, *Streptococcus mutans*, with *L. salivarius* having the greatest potency (Wasfi et al., 2018), an effect that was ascribed to decreased expression of genes involved in exopolysaccharide synthesis, acid tolerance, and quorum sensing (Wasfi et al., 2018).

*Escherichia coli* Nissle 1917 has been used as a probiotic for many years to treat various intestinal disorders. Hancock et al. (2010) reported that this bacterium can outcompete pathogenic *E. coli* strains such as enteropathogenic and enterotoxigenic *E. coli* during biofilm formation and it has been reported that *E. coli* Nissle can inhibit growth, gas production and toxin production (α-toxin and NetB) by *C. perfringens* in a cell-dependent manner (Jiang et al., 2014).

The anti-biofilm effects of various candidate probiotics have also been studied against upper respiratory tract pathogens. A mixture of *Streptococcus salivarius* and *Streptococcus oralis* is reported to significantly reduce biofilm formation by *S. aureus*, *S. epidermidis*, *S. pneumoniae*, *P. acnes* and *M. catarrhalis* (Bidossi et al., 2018). Similar effects of *Bifidobacterium longum* have been reported against EHEC O157:H7, with the autoinducer (AI-2) activity being identified as the target (Kim et al., 2012).

The potential inhibitory effect of lactobacilli on the formation of biofilms by the food-borne pathogens *Salmonella enterica* serovar Typhimurium and *Listeria monocytogenes* has been investigated, where it was reported that *L. rhamnosus* and *L. paracasei* both significantly reduced biofilm formation by *L. monocytogenes* by competing with, excluding, and displacing pathogenic bacteria (Woo and Ahn, 2013). The study also reported that only *L. paracasei* significantly displaced the cells in the *S.* Typhimurium biofilms, but that *S.* Typhimurium was significantly diminished by *L. acidophilus* due to competition. In a study on the voice prostheses biofilms, it was reported that treating prostheses in an artificial throat model with biosurfactants derived from *Lactococcus lactis* and *Streptococcus thermophiles* led to a significant reduction in the number of bacteria and subsequently reduced the airflow resistance. This may be promising for extending the lifespan of voice prostheses (Rodrigues et al., 2004).

We hypothesized that a wide range of bacteria may produce factors that inhibit various aspects of microbial virulence. The aim of the current study was therefore to investigate the antimicrobial and antibiofilm activities of a range of bacteria including organisms isolated from human skin, and the candidate probiotic *E. coli* Nissle 1917. *E. coli* Nissle 1917 is a commonly studied candidate probiotic bacterium. *P. aeruginosa* was used as a representative versatile pathogen.

## Materials and methods

### Bacterial strains and growth conditions

The *Pseudomonas aeruginosa* strains used in this study were PAO1 along with SNP0614, BR1-B, LYT-4 which were wound isolates. *Escherichia coli* Nissle 1917 was isolated from Mutaflor tablets, manufactured by Ardeypharm (Herdecke, Germany). Comparator *Escherichia coli* strains were *E. coli* WIBG 2.4 (Oates et al., 2014) as well as *E. coli* K-12 MG165 and *E. coli* ATCC 25922. The human commensal bacteria that were tested are listed in Supplementary Table S1. All organisms were grown in Luria agar or broth and incubated aerobically at 37°C.

### Chemicals and media

All chemicals and dehydrated bacteriological media were provided by (Sigma-Aldrich, United Kingdom). Petri dishes, 96-well microtiter plates and different reagents were purchased from (Sigma-Aldrich, United Kingdom). Tips, microcentrifuge tubes, solutions and growth media were sterilized in an autoclave at 121°C for 30 min.

### Preparation of bacterial supernatants

Stationary phase broths (10 ml) of the test bacteria were harvested by centrifugation at 4,000 rpm for 10 min. The supernatant of the bacterium was then passed through 0.22 μm Millex-GV syringe filters to remove any resident cells.

### Antibacterial assay

The methodology was adapted from Wilson et al. (2021). An overnight broth culture of *P. aeruginosa* was adjusted spectrophotometrically to OD600 of 0.8 and then diluted 1:100 and a total of 200 μl (100 μl of the prepared broth and 100 μl of tested bacterial cell-free supernatant, 50% *v*/*v*) was inoculated into 96 well plates. Control wells were inoculated with 100 μl of each pathogen together with 100 μl of its supernatant. The plate was then incubated in a Powerwave XS plate reader (Biotek, Bedfordshire, United Kingdom) at 37°C, where the absorbance of each well was measured at 600 nm every 1 h over 24 h to determine the effect of the cell-free supernatant on the growth rate of *P. aeruginosa*. The growth curves were analyzed using the Gen5 Software program (Biotek, Bedfordshire, United Kingdom). The experiment was run in triplicate and repeated on at least three separate occasions for each organism.

### Biofilm inhibition assay

The crystal violet biofilm assay procedure was adapted from O’Toole (2011). This was used to assess the effects of cell-free supernatant of bacteria on biofilm formation by *P. aeruginosa*. An overnight broth of *P. aeruginosa* was adjusted to OD600 of 0.8 and then diluted 1:100, and 100 μl of the prepared broth was inoculated into the wells of 96 well plates with peg lids (Calgary devices) with 100 μl of the tested bacterial cell-free supernatant. In this way, the cell-free supernatant was diluted 1:2 with a final concentration of 50% *v*/*v*, and 100 μl of *P. aeruginosa* was inoculated with 100 μl of its supernatant used as a negative control. The compartment was then covered with the sealed peg lid and the plate was incubated for 24 h at 37°C. After 24 h, the sealed peg lid containing the biofilm was transferred first to a new 96-well plate containing PBS to wash any unattached bacteria (planktonic cells) and then the attached biofilms were stained with 200 μl of filtered 1% (*w*/*v*) crystal violet and washed with PBS to wash away any excess stain. Finally, the pegs were immersed in a new well plate containing 200 μl of absolute ethanol and the absorbance of the de-stained crystal violet was measured using a plate reader at 590 nm. The percentage of biofilm inhibition was calculated using the following formula: (%) = [(Control OD 590 nm − Treated OD 590 nm)/Control OD 590 nm] × 100.

### Minimal biofilm inhibitory concentration

The MBIC was determined for Nissle cell-free supernatants using a method modified from a previously described procedure (Yang et al., 2021). Nissle cell-free supernatants were serially diluted two-fold using the supernatant of the pathogen as diluent. The cell-free supernatants were diluted in the range of 50–3% (*v*/*v*). Subsequently, each concentration was co-incubated with the pathogen and tested for biofilm inhibition. The anti-biofilm activities of the different concentrations of the supernatant were compared using crystal violet biofilm assay against *P. aeruginosa*. The experiment was performed in triplicate.

### Biofilm disruption assay

The crystal violet biofilm assay (O’Toole, 2011) was performed to assess the effect of Nissle cell-free supernatant on mature *P. aeruginosa* biofilms. An overnight broth of each bacterium was adjusted to an OD600 of 0.8 and then diluted 1:100. Aliquots of 200 μl of the prepared broth were inoculated into the wells of 96 well plates with Calgary device peg lids, and incubated for 24 h at 37°C to allow biofilm formation. Subsequently, the peg lid containing the biofilm was aseptically washed with PBS to remove any unattached bacteria (planktonic cells) then the attached biofilms were moved to another 96 well plates containing 200 μl of Nissle cell-free supernatant. *P. aeruginosa* supernatant was used as a control. The plates were incubated for a further 24 h at 37°C. After incubation periods, the biofilm was transferred to a new 96-well plate containing PBS. The attached biofilms were then stained with 200 μl of filtered 1% (*w*/*v*) crystal violet and washed with PBS. Finally, the pegs were immersed in a new well plate containing 200 μl of absolute ethanol and the absorbance of the de-stained crystal violet was measured using a plate reader at 590 nm. The percentage of biofilm dispersion was calculated using the following formula: (%) = [(Control OD 590 nm − Treated OD 590 nm)/Control OD 590 nm] × 100.

### Confocal microscopy of *Pseudomonas aeruginosa* biofilms

*Pseudomonas. aeruginosa* cultures incubated for 24 h were adjusted to an OD600 of 0.8 and then diluted 1:100, and 1 ml of the prepared broth was inoculated into ibiTreat petri dish (ibidi, Munich, Germany) with 1 ml of the Nissle cell-free supernatant. The dish was incubated for 24 h at 37°C. 1 ml of *P. aeruginosa* inoculated with 1 ml of its supernatant was used as a negative control. For assessing effects on mature biofilms, an overnight broth of *P. aeruginosa* was adjusted to OD600 of 0.8 and then diluted 1:100 and 1 ml of the prepared broth was inoculated into u-Dish (ibidi) and incubated for 24 h at 37°C. After incubation, the planktonic cells were removed and 1 ml of Nissle cell-free supernatant was added and then incubated for another 24 h. 1 ml of *P. aeruginosa* supernatant was used as a negative control.

### Confocal microscopy analysis of biofilm inhibition

Biofilms were stained with the cell-impermeant nucleic acid stain TOTO-1 (2 μM for 5 min., at 37°C; Thermo Fisher Scientific, MA, United States) and counterstained with the cell-permeant nucleic acid stain SYTO-60 (10 μm for 15 min, at 37°C; Thermo Fisher Scientific, MA, United States). Samples were visualized using excitation lasers of 488 nm and 640 nm for TOTO-1 and SYTO-60, respectively. An emission band of 450–630 nm was acquired for TOTO-1 and an emission band of 656–700 nm was acquired for SYTO-60. Biofilms were observed on a Leica SP8 Inverted Tandem Head confocal microscope using a 63 × NA 1.4 oil immersion objective and a 1 × confocal zoom. The images were collected sequentially to eliminate crosstalk between channels. When acquiring Z-stack, confocal software was used to determine the optimal number of Z sections. 3D optical stack reconstruction of biofilms and quantification were performed using Imaris 9.0 software (Bitplane, Zurich, Switzerland).

### Physicochemical characterization of *Escherichia coli* Nissle cell-free supernatant

The methodology was adapted from (Yang et al., 2021). Aliquots of the supernatant were treated with different enzymes (proteolytic and non-proteolytic): lipase, 𝛼-amylase, and proteinase K. Enzyme-treated supernatants were activated by incubating at 37°C for 3 h, after which the enzymes were immediately inactivated at 95°C for 3 min. Untreated supernatant was used as control. The heat stability of the supernatant was also evaluated by incubating aliquots of the supernatant at different temperatures of 50, 70, and 100°C for 1 h. For each of the above tests, the anti-biofilm activities of treated and untreated culture supernatants were compared using crystal violet biofilm assay against *P. aeruginosa*. Each data point is composed of three independent experiments performed in at least three replicates.

### Size assessment of active fractions

The supernatant was separated with different molecular weight cut-offs (MWCO) using a protein concentrator (Amicon Ultra centrifugal filter units, Merck, Germany) *via* centrifugation at 14000 × *g*. The supernatants (MW < 30 MW 30 ~ 50 MW 50 ~ 100 MW > 100 kDa) were collected as a flow-through using 30, 50, and 100 K MWCO concentrators. The concentrated and un-filtrated supernatants were diluted with PBS to restore their volume to be the same as the initial volume. Each fraction was tested on *P. aeruginosa* biofilms using a crystal violet biofilm assay.

### *Galleria mellonella* pathogenicity assay

This was performed as described previously (Moman et al., 2020). Larvae of *G. mellonella* were incubated for 30 min at room temperature before injection. Overnight cultures of *P. aeruginosa* and *E. coli* Nissle were centrifuged (4,000 rpm, 10 min) and suspended in PBS. This was repeated twice. Cultures were adjusted to an OD600 nm of 0.1 for intrahemocoelic injection, bacterial suspensions were prepared with final concentrations in the range of 10^4^ CFU/ml to 10^8^ CFU/ml. *E. coli* Nissle viable cells and the supernatant were administrated to the larvae either simultaneously with *P. aeruginosa* or 24 h before the injection with the pathogen. Volumes of 5 μl of each bacterium or cell-free supernatant were delivered directly to the hemocoel through an injection in the rear left pro-leg using a 26-gage needle Hamilton microsyringe (Sigma, United Kingdom). Sterile PBS (5 μl) was injected into the trauma control group and additionally, a no-treatment control group was added. The right pro-leg was used as the injection site. Different sites were used for the pathogen and the probiotic or its supernatant to reduce the risk of injection site infection. Infected larvae were incubated in a petri dish in groups of 10 at 37°C in the dark for 7 days. Larval mortality was determined daily over a week. Larvae that had turned black and that were not moving in response to a gentle shaking of the dish or touching with a pipette tip were considered dead. Dead larvae were removed from the petri dish and the death was recorded. The experimental endpoint was designated by either the death of all the larvae in the tested groups or the conversion of larvae into pupae. Pupae were identified *via* a color change to white (Jorjão et al., 2018). Three Petri dishes containing ten larvae each were assigned to each experiment and control group (30 larvae total for each sample). The experiments were terminated once two of the control individuals had died or pupated.

### Proteomic analysis of *Pseudomonas aeruginosa* biofilms

#### Extraction of proteins

*Pseudomonas aeruginosa* was grown in Luria broth overnight at 37°C. The overnight culture OD was adjusted to 0.8 then diluted 1:100 and 2 ml of the prepared broth was inoculated into a 24-well plate with or without the presence of Nissle cell-free supernatant. The plate was then incubated for 24 h at 37°C to allow biofilm formation. The bacterial biofilms were aseptically transferred into tubes and the biofilm cells were collected by centrifugation at 1500 × *g* for10 min at 4°C. the supernatant was discarded, and the pellet was washed twice with PBS and centrifuged at 1500 × *g* for 10 min at 4°C. The PBS was removed, and the pellets were weighed on a fine balance to add 3 μl of lysis buffer (0.05% SDS in 200 mM Tris–HCL pH 8.0) for every 1 mg of the pellet and sonicated for 6 min on ice. The protein concentration was measured in each sample using bicinchoninic acid (BCA) assay and 30 μg of the protein lysate was used for peptide digestion. To reduce the protein lysate, a solution of DTT (prepared in 20 mM AMBIC) was added to 30 ug protein lysate at a final concentration of 10 mM and incubated at 60°C in an incubator shaker for 30 min. Reduced cysteine residues were alkylated by adding Iodoacetamide solution (prepared in 20 mM AMBIC) at a final concentration of 50 mM and incubation proceeded for 30 min at room temperature, in the dark. Another reduction was carried out by adding a solution of DTT (prepared in 20 mM AMBIC) at a final concentration of 10 mM and incubated for 10 min at room temperature, in the dark. Mass spectrometry grade trypsin was added in a 1:50 (enzyme: protein). The digestions took place overnight at 37°C while shaking. A second digestion in 80% Acetonitrile was performed by adding trypsin in a 1:100 (regardless of the protein content of the sample) and added 80% (*v*/*v*) Acetonitrile in the final sample volume and incubated at 37°C for 3 h. Trypsin activity was stopped by adding 5% (*v*/*v*) formic acid. The digested samples were dried to completion using a vacuum centrifuge and then stored at –20°C until analysis.

#### LC–MS/MS protein analysis

The extracted samples were resuspended in 0.1% formic acid and 1 μg portions were analyzed by LC–MS/MS using a U3000 NanoRSLC liquid chromatography system (ThermoScientific), coupled to an Orbitrap Fusion Lumos Tribrid (ThermoScientific, San Jose, CA, United States). Peptides were previously concentrated on a trap column (C18 5 μm 0.1 × 20mm at 5 μl/min) and separated on an EASYSPRAY PEPMAP RSLC (C18 2 μm particle size, 50 cm × 75 μm i.d.; Thermo Fisher Scientific) using a 135-min method at a flow rate of 300n/min. For 5 min at 3% B, 5-125 min from 3 to 35%B, 125–125.5 min from 35 to 95%B, 125.5-128 min at 95%B, 128–128.5 min from 95 to 3%B, from 128.5 to 135 min at 3%B. Solvents were composed of: A: 0.1% formic acid, B: 80% acetonitrile/0.08% formic acid). The peptides were ionized using an electrospray nanoEasySpray ion source. The mass spectrometer was operated in Date Independent Acquisition (DIA) mode. The method was divided into two sections. In the first section, the mass of the peptides was measured using an orbitrap at a resolution of 120,000, with an m/z range of 375–1,500, the maximum injection time was set to 50 ms and the normalized AGC target to 100%. The data were acquired in positive profile mode. In the second section of the DIA method, peptides in a defined m/z interval were fragmented using HCD at 35% and the mass of the fragments was measured using an orbitrapat 30,000 resolution with a maximum injection time set to 54 ms and normalized AGC target set to 100%. The data were acquired in positive centroid mode.

### Statistical analyses

All data are presented as the mean ± SEM of at least three independent experiments with triplicate samples within each independent experiment. All statistical tests were carried out using GraphPad Prism v9·0 software (GraphPad Software Inc., La Jolla, CA, United States). Data were analyzed by One-way ANOVA and differences were considered statistically significant at a value of *p* < 0.05. *G. mellonella* data were plotted as survival curves using the Kaplan–Meier estimator.

## Results

### Cell-free supernatants of tested bacteria did not inhibit the growth of *Pseudomonas aeruginosa* in planktonic culture

**Figure 1:**
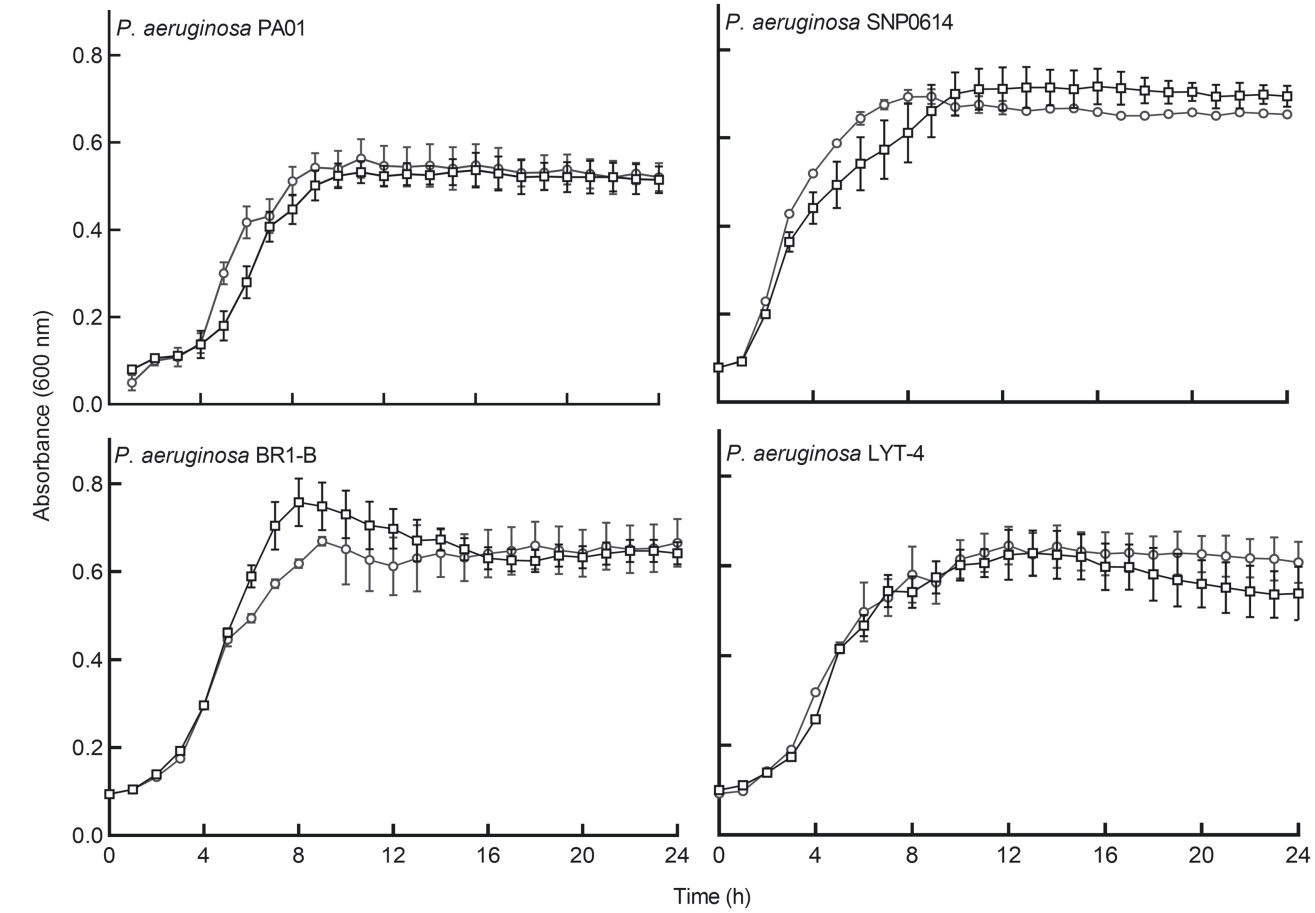
*E. coli* Nissle cell-free supernatant did not significantly inhibit the planktonic growth of four strains of *P. aeruginosa*. Circular symbols, co-incubation of *Pseudomonas aeruginosa* strains with Nissle supernatant. Square symbols, control cultures. Data are mean ± SEM, *n* = 3. Significance was set at *p* < 0.05.

We determined whether cell-free supernatants of the bacterial isolates contained antibacterial substances. The supernatants showed no inhibitory effects on the planktonic growth of *P. aeruginosa* in a microtiter plate when incubated for 24 h. Cell-free supernatants of *E. coli* Nissle 1917 were also tested for antibacterial activity against different *P. aeruginosa* strains. *E. coli* Nissle cell-free supernatants did not have any inhibitory effects on the growth rate of any of the tested *P. aeruginosa* strains (Figure 1).

### *Escherichia coli* Nissle cell-free supernatants inhibit biofilm formation by *Pseudomonas aeruginosa*

**Figure 2:**
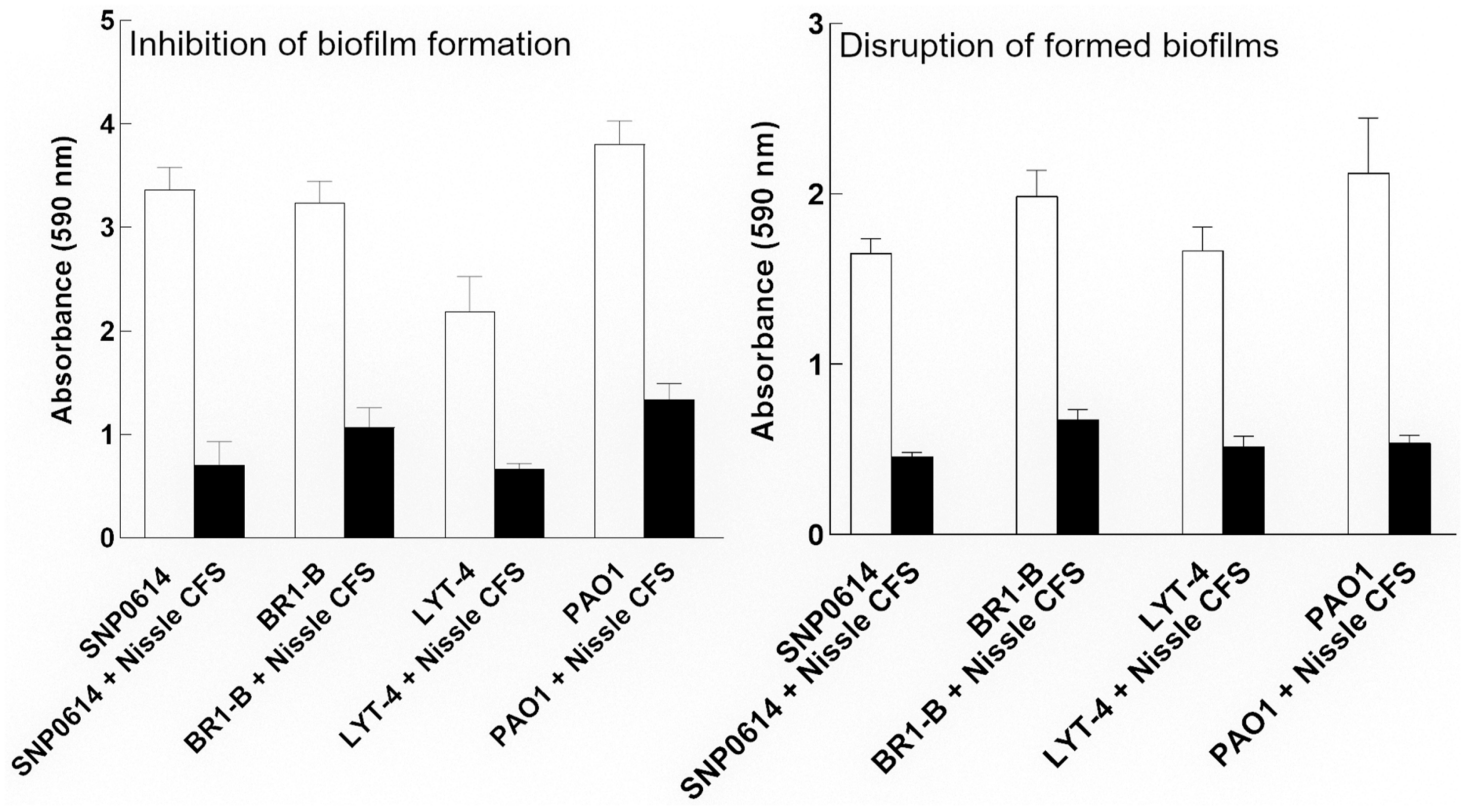
Relative biofilm formation of *P. aeruginosa* (PA) strains grown in the presence of Nissle cell-free supernatant (CFS) measured by crystal violet staining. Nissle cell-free supernatant significantly inhibited biofilm formation and disrupted mature biofilms for all tested *P. aeruginosa* strains. Data are mean ± SEM., *n* = 3.

We subsequently determined whether cell-free supernatants of the tested bacteria had any biofilm inhibitory activities against *P. aeruginosa*. Cell-free supernatants of most of the bacterial isolates did not show antibiofilm effects on *P. aeruginosa* when incubated in a microtiter plate for 24 h. However, *E. coli* Nissle cell-free supernatant significantly inhibited biofilm formation by all *P. aeruginosa* strains (Figure 2A). To assess whether the antibiofilm activity is unique to *E. coli* Nissle, *E. coli* reference strains ATCC 25922, K-12 MG165 and a wound isolate (WIBG 2.4) were tested. The supernatants of these bacteria did not show any biofilm-inhibitory effects against *P. aeruginosa* (Supplementary Figure S2).

### *Escherichia coli* Nissle cell-free supernatant disperses mature biofilms of *Pseudomonas aeruginosa*

We subsequently, investigated whether Nissle cell-free supernatant can disperse formed biofilm of *P. aeruginosa* strains. For that, the strains were allowed to adhere to the surface of a microtiter plate and form biofilms for 24 h. The formed biofilms were co-incubated with Nissle cell-free supernatant to check for disruption activity. The supernatant was significantly able to disrupt the mature biofilm produced by all strains after 24 h incubation (Figure 2B). Viable cell counts of mature *P. aeruginosa* biofilms treated with or without Nissle cell-free supernatants were also investigated by plate counting. The results showed no significant difference in biofilm cell counts between the control and the treated samples (Supplementary Figure S3).

### Dose–response in the inhibition of *Pseudomonas aeruginosa* biofilm formation by *Escherichia coli* Nissle cell-free supernatants

**Figure 3:**
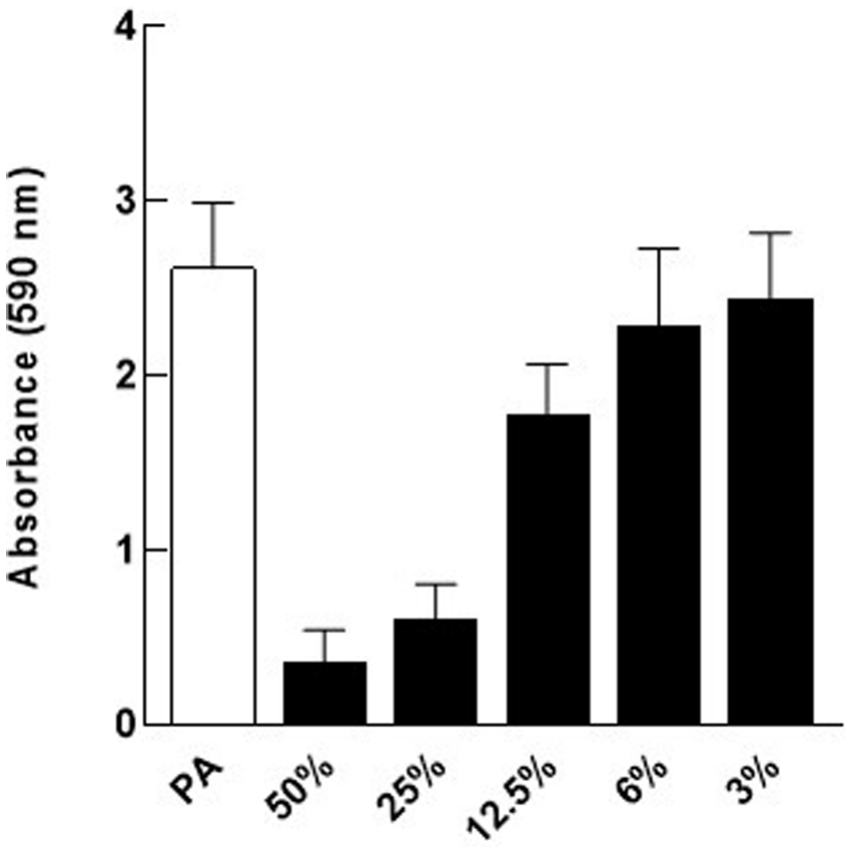
Dose response for Nissle cell-free supernatant (CFS) on *P. aeruginosa* (PA) biofilm. Different dilutions of Nissle cell-free supernatant were tested on *P. aeruginosa* biofilm to determine the minimal biofilm inhibitory concentration (MBIC). 25% (*v*/*v*) cell-free supernatant was the lowest concentration that caused significant biofilm inhibition against *P. aeruginosa*. Data are mean ± SEM, *n* = 3.

To determine the lowest concentration of cell-free supernatant that could inhibit biofilm formation of *P. aeruginosa*, two-fold dilutions of cell-free supernatant ranging between 50 and 3% (*v*/*v*) were tested. Data indicate that 25% (*v*/*v*) was the lowest concentration that retained significant biofilm inhibitory activity (Figure 3).

### *Escherichia coli* Nissle cell-free supernatant inhibits the accumulation of eDNA and disperses formed eDNA in *Pseudomonas aeruginosa* biofilm

**Figure 4:**
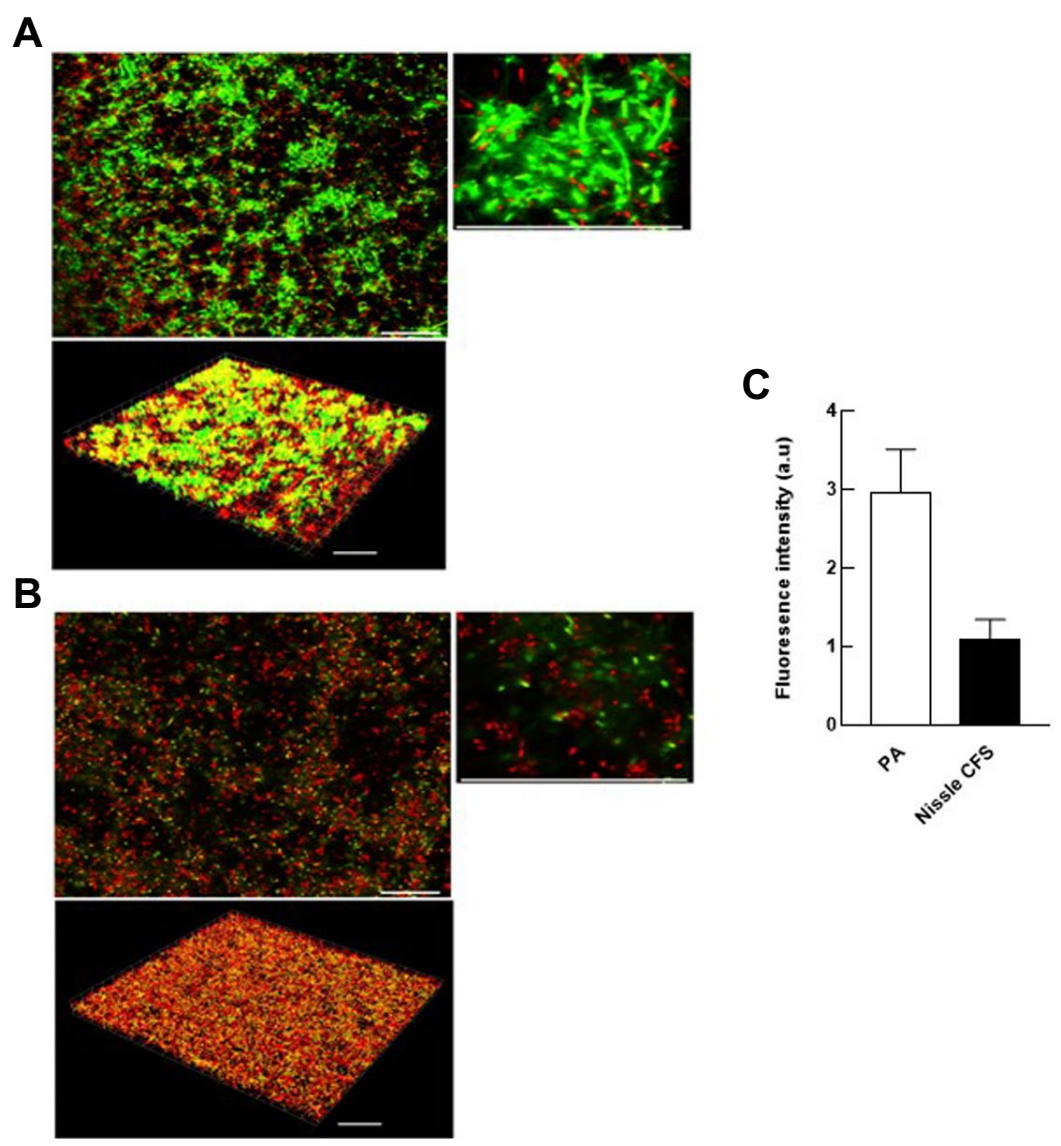
*E. coli* Nissle cell-free supernatant (CFS) reduces the accumulation of eDNA in developing biofilms. 2D and 3D confocal images of *P. aeruginosa* (PA) biofilms grown in the absence **(A)** and presence **(B)** of cell-free supernatants of *E. coli* Nissle. Biofilms were stained with the eDNA-specific cell-impermeant nucleic acid stain TOTO-1 (Green) and counterstained with a cell-permeant nucleic acid stain, SYTO-60 (Red). Viable cells appear red while eDNA appears green. Dead cells appear are yellow (co-localization of red and green color). **(C)** Quantification of fluorescence signals derived from CLSM 3D imaging indicates that the accumulation of eDNA of the matrix of *P. aeruginosa* biofilms was significantly reduced by incubation with Nissle cell-free supernatants. Data are means ± SEM (*n* = 3). Scale bars = 30 μm.

**Figure 5:**
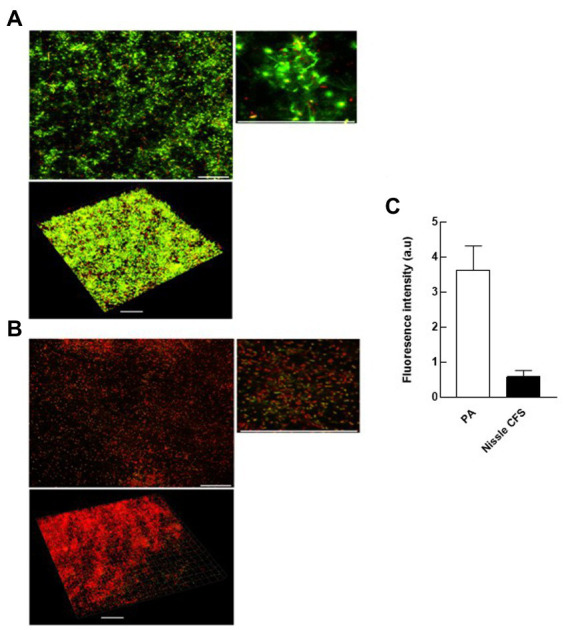
*E. coli* Nissle cell-free supernatant (CFS) reduces eDNA in extant biofilms. 2D and 3D confocal images of *P. aeruginosa* (PA) biofilms grown in the absence **(A)** and presence **(B)** of cell-free supernatants of *E. coli* Nissle. **(C)** Quantification of fluorescence signals derived from CLSM 3D imaging. See legend to Figure 4.

Confocal laser scanning microscopy (CLSM) was used to visualize the changes in eDNA by staining with TOTO-1 to detect eDNA and dead cells, and Syto-60 to detect live cells of *P. aeruginosa* biofilm matrix before and after treatment with *E. coli* Nissle cell-free supernatant. We found that 24 h incubation of *E. coli* Nissle cell-free supernatant with *P. aeruginosa* significantly inhibits the accumulation of eDNA of the matrix in *P. aeruginosa* biofilm. Moreover, we also observed that the cell-free supernatant of *E. coli* Nissle significantly reduced the eDNA signal in 24 h formed *P. aeruginosa* biofilm after treatment with *E. coli* Nissle cell-free supernatant. The results obtained from the confocal images suggest that the cell-free supernatant of *E. coli* Nissle reduced the accumulation of eDNA (Figure 4) and degraded formed eDNA on the *P. aeruginosa* biofilms (Figure 5). eDNA fluoresced green dead cells yellow signal and live cells red (Afshar et al., 2022).

### Proteinaceous compounds are involved in anti-biofilm activities

**Figure 6:**
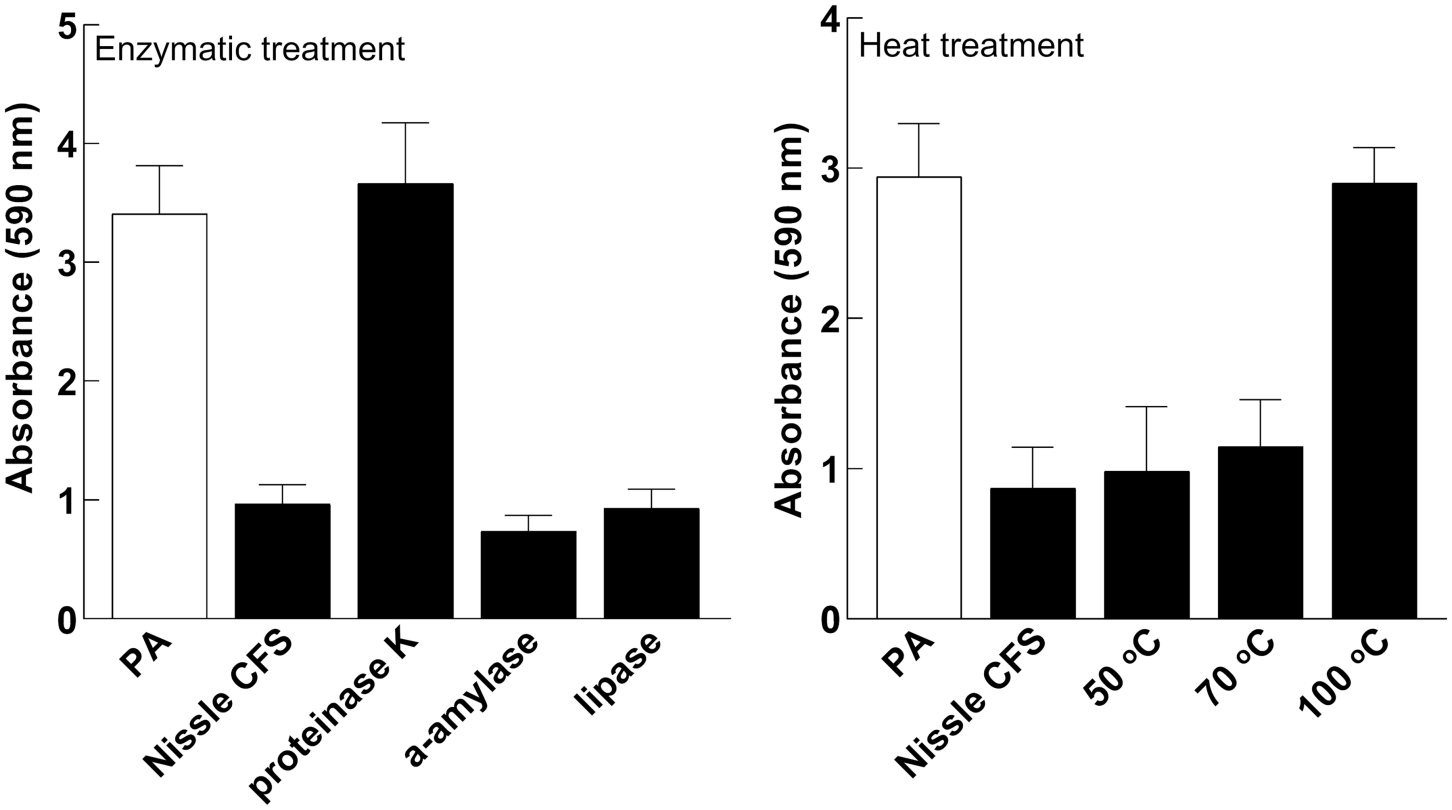
The active factor is proteinaceous and heat-labile. Effects of Nissle cell-free supernatants (CFS) on *P. aeruginosa* (PA) biofilm after enzymatic and heat treatment. Proteinase K eliminated the inhibitory effects. Lipase or α-amylase did not. The anti-biofilm activity was inhibited by heating to 100°C. Data are mean ± SEM, *n* = 3.

To characterize components responsible for the activity of *E. coli* Nissle cell-free supernatant against *P. aeruginosa*, the antibiofilm effects of cell-free supernatants were evaluated against *P. aeruginosa* following incubation with Proteinase K, Lipase, and α-amylase. Treatment of cell-free supernatants with Proteinase K eliminated the inhibitory effects of the cell-free supernatant on *P. aeruginosa* biofilm, while Lipase or α-amylase did not (Figure 6A). The antibiofilm component was also inactivated by heating to 100°C (Figure 6B).

### The active fraction of the antibiofilm in *Escherichia coli* Nissle cell-free supernatant is greater than 30 kDa in size

**Figure 7:**
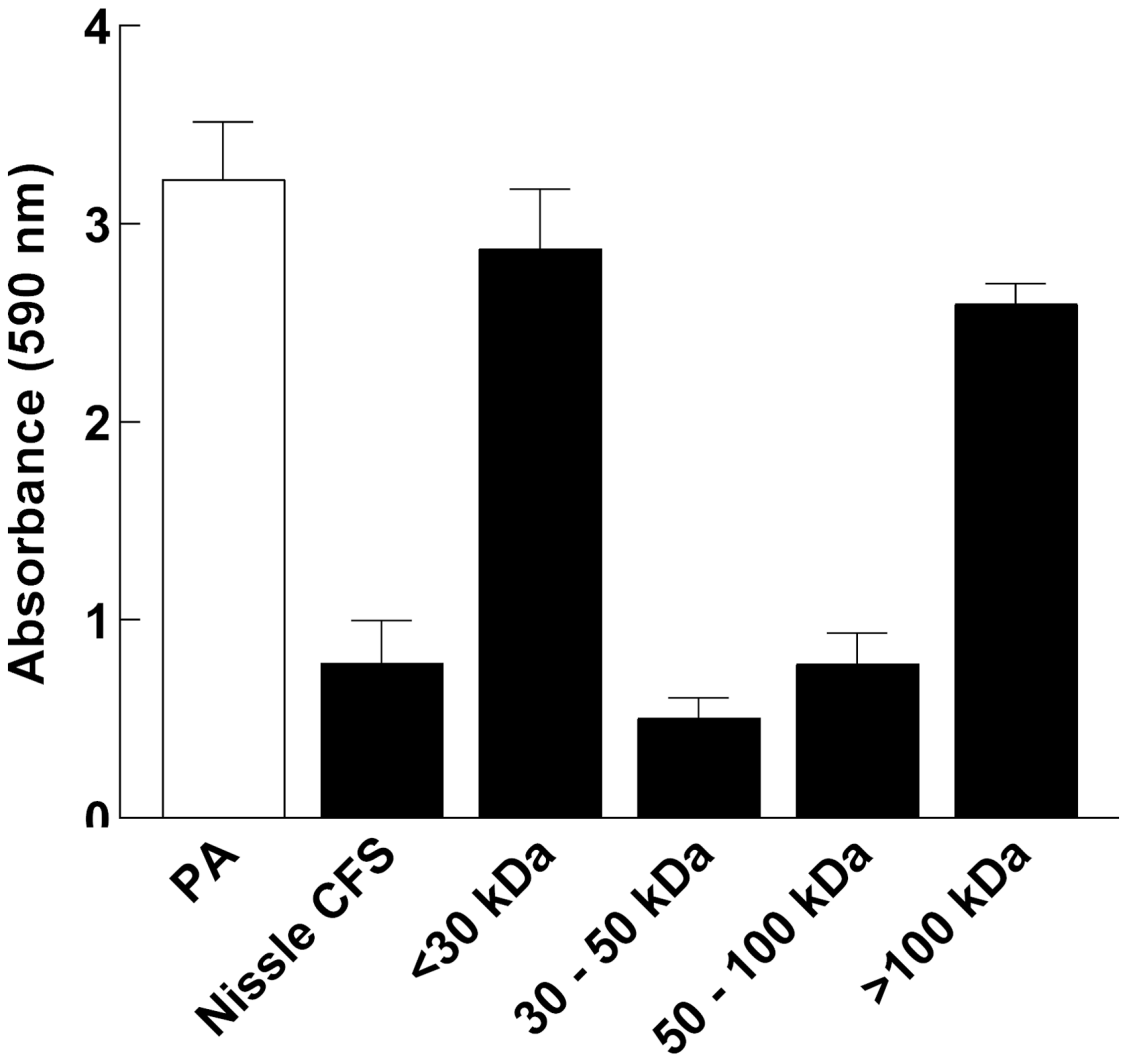
*P. aeruginosa* (PA) biofilm formation was significantly inhibited by 30 and 100 kDa fractions of Nissle cell-free supernatant (CFS), while there was no inhibition observed in fractions lower than 30 kDa and higher than 100 kDa. Aliquots of cell-free supernatant were separated with different molecular sizes and each fraction was tested against biofilm formation by *P. aeruginosa*. Whole cell-free supernatant was used as a control. Data are mean ± SEM, *n* = 3.

Cell-free supernatant was separated by size fractionation using columns with different size cut-offs (30, 50, and 100 kDa) and each fraction was tested against *P. aeruginosa* biofilm in a microtiter plate assay. *P. aeruginosa* biofilm formation was significantly inhibited by 30 and 100 kDa fractions, while there was no inhibition observed in fractions lower than 30 kDa and higher than 100 kDa which indicates that the size of the antibiofilm component is between 30 and 100 kDa (Figure 7).

### *Escherichia coli* Nissle cell-free supernatant protects *Galleria mellonella* infected with *Pseudomonas aeruginosa*

**Figure 8:**
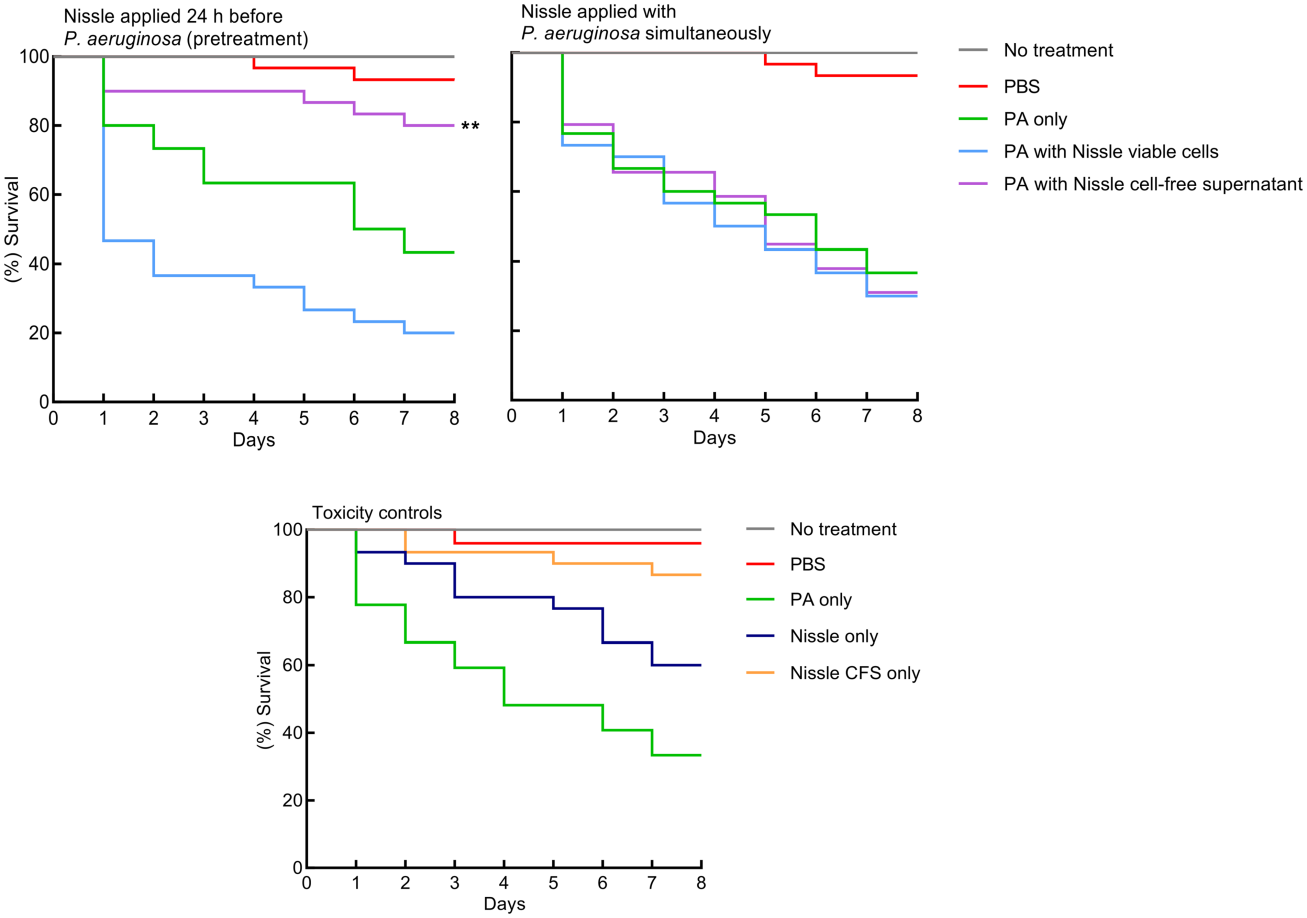
Pretreatment with *E. coli* Nissle cell-free supernatant (CFS) confers significant protection against *P. aeruginosa* toxicity in a *Galleria mellonella* virulence assay. Kaplan–Merier survival curves of larvae injected with Nissle cells or supernatants 24 h before, or simultaneously with *P. aeruginosa*. Pre-treatment with Nissle cell-free supernatant conferred significant protection to the larvae. Administration of Nissle viable cells increased larval mortality. *P. aeruginosa* was significantly lethal to the larvae. No significant toxic eﬀects of the Nissle cell-free supernatant were observed. Asterisks indicate statistically significant diﬀerences (*p* < 0.05).

The protective effects of viable cells of *E. coli* Nissle or cell-free supernatants on larvae injected with *P. aeruginosa* were evaluated. Initially, *P. aeruginosa*, *E. coli* Nissle viable cells and its cell-free supernatant were injected separately into the larvae to determine toxicity. *P. aeruginosa* was significantly lethal to the larvae. *E. coli* Nissle viable cells were less lethal to the larvae and no significant toxic effects of its cell-free supernatant were observed on the larvae compared to the untreated group (Figure 8). *Escherichia coli* Nissle viable cells or cell-free supernatants were then administrated to the larvae either simultaneously or 24 h before the pathogen challenge. Simultaneous administration of both Nissle live cells and its cell-free supernatants with the pathogen did not increase larvae survivability (Figure 8). However, Nissle cell-free supernatants were administrated 24 h before pathogen injection. This conferred significant protection to the larvae against the pathogen. However, pre-treatment with *E. coli* Nissle viable cells increased the mortality of the larvae (Figure 8).

### *Escherichia coli* Nissle cell-free supernatant downregulates essential proteins related to biofilm formation, and other virulence factors in *Pseudomonas aeruginosa*

**Figure 9:**
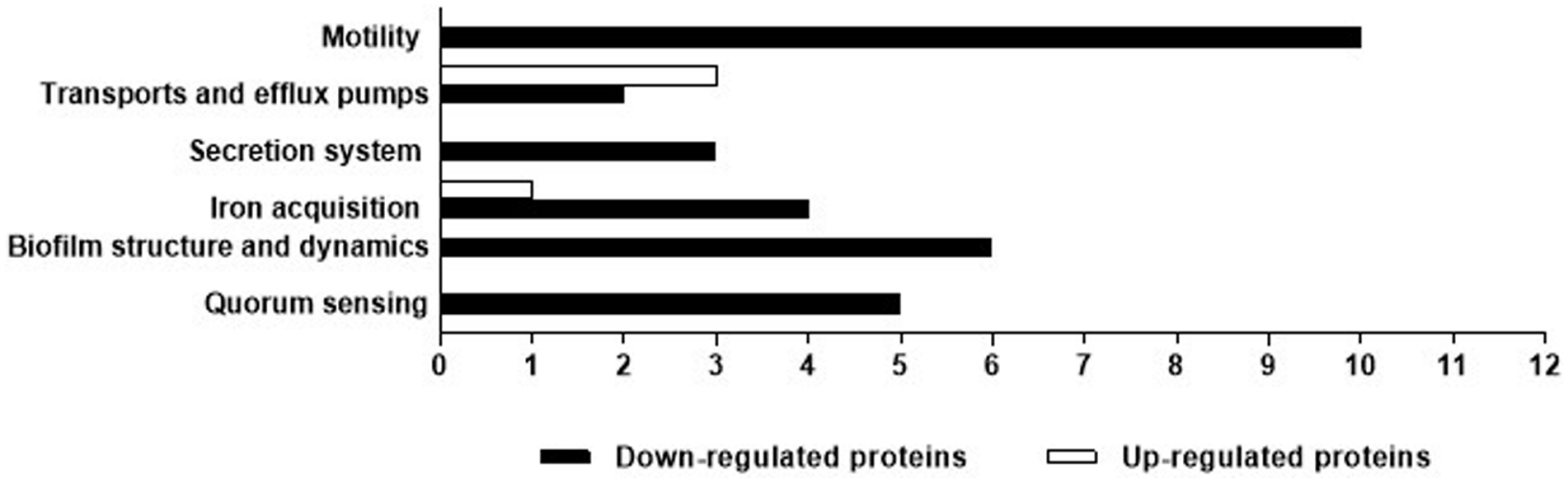
*P. aeruginosa* biofilm-related proteins and other virulence factors that had a significant (> 1.5-fold change) difference in production between the control and Nissle cell-free supernatant. Proteins were classified according to their function.

Biofilm-related proteins and other virulence proteins that were up or downregulated (> 1.5-fold change) in the presence of cell-free supernatant were investigated (Table 1; Figure 9). Proteomic analyses revealed that some biofilm-related proteins and other virulence factors were differentially expressed between control and cell-free supernatant-treated samples. Motility-related proteins (i.e., flagellum, flagella, and type IV pili) proteins were generally downregulated by at least a 1.5-fold change with the presence of CFS. Also, the quorum-sensing molecule acyl-homoserine lactone synthase lasI as well as HTH-type quorum-sensing regulator rhlR were downregulated (> 2.6- > 8.5-fold) respectively. In addition, rhamnosyltransferase subunits, i.e., RhlA and RhlB were significantly downregulated (> 3.6- > 15-fold) respectively. Moreover, upon treatment with cell-free supernatant, the expression level of RNA polymerase sigma factor RpoS and positive alginate biosynthesis regulatory protein algR, alginate biosynthesis sensor protein KinB were all significantly reduced ( > 5- > 3.7 and > 2.2-fold) respectively compared to the control samples. However, Elastase LasB was not significantly expressed between treatments and controls. Furthermore, Pyoverdine and Pyochelin were shown to be differently expressed between control and treatment groups, i.e., L-ornithine N (5)-monooxygenase pvdA and Pyochelin synthase PchD, PchF were significantly downregulated (> 37- > 3.4- > 6.7-fold) respectively. In contrast, Fe (3+)-pyochelin receptor fptA was upregulated (> 6.2-fold) after being treated with cell-free supernatant. Moreover, type II secretion system-related proteins xcpS and xcpT were significantly downregulated (> 30 and > 4-fold) respectively. In addition, Type VI secretion system sheath protein TssC1 was downregulated by > 13 times compared to the control samples. Transport and efflux pumps-related proteins, on the other hand, were differentially expressed upon treatment with *E. coli* Nissle cell-free supernatant, i.e., outer membrane porin D oprD, outer membrane protein assembly factor BamD, and the outer membrane lipoprotein oprI were upregulated by (> 10.8– > 3.8– > 2.6) respectively. However, the outer membrane porin F oprF, and TonB were downregulated (> 5.1–> 15.3) respectively.

**Table 1:** *Pseudomonas aeruginosa* biofilm-related proteins and other virulence factors that were significantly expressed (> 1.5-fold change) after challenging with Nissle cell-free supernatant.

Protein accession	Gene name	Protein description	Fold change	*p*-Value
Motility				
P22608	pilB	Type IV pilus assembly ATPase PilB	-3.24404	0.001195
P34750	pilQ	Fimbrial assembly protein PilQ	-2.520677	0.027357
P72151	fliC	B-type flagellin	-17.47914	0.0021
Q51466	fliN	Flagellar motor switch protein FliN	-3.623068	0.049282
Q9I4N6	fliS	Flagellar secretion chaperone FliSB	-45.20697	0.002183
Q9K3C5	fliD	B-type flagellar hook-associated protein 2	-2.954447	0.017396
Q9HZA6	fimV	Motility hub protein FimV	-3.789192	0.003654
G3XD28	pilM	Type IV pilus inner membrane component PilM	-7.897769	0.00027
G3XD30	pilN	Type IV pilus inner membrane component PilN	-5.039217	0.0012
G3XD64	fleN	Antiactivator FleN	-2.586525	0.013791
Quorum sensing				
P33883	lasI	Acyl-homoserine-lactone synthase	-2.644369	0.007434
Q9I4X1	pqsC	2-heptyl-4(1H)-quinolone synthase subunit PqsC	-9.559499	0.00382
Q9I4X3	pqsA	Anthranilate--CoA ligase	-11.73713	0.000264
P54291	rhlI	Acyl-homoserine-lactone synthase	-16.23003	0.0074562
P54292	rhlR	HTH-type quorum-sensing regulator RhlR	-8.542291	0.000018
Biofilm structure and dynamics				
P26275	algR	Positive alginate biosynthesis regulatory protein	-3.704029	0.0182576
O34206	kinB	Alginate biosynthesis sensor protein KinB	-2.238969	0.0000374
O33407	estA	Esterase EstA	-10.93847	0.00759
Q9HXE5	rhlB	Rhamnolipid	-15.00906	0.0326822
Q51559	rhlA	Rhamnolipid	-3.660112	0.000439
P45684	rpoS	RNA polymerase sigma factor RpoS	-4.979014	0.000375
Iron acquisition				
Q51548	pvdA	L-ornithine N (5)-monooxygenase	-37.33491	0.0056734
P42512	fptA	Fe (3+)-pyochelin receptor	6.182236	0.0000372
Q9HWG	pchD	Pyochelin synthase PchD	-3.483494	0.00018
G3XCV2	pchE	Pyochelin synthase PchE	-2.809508	0.049381
Q9HY07	pchF	Pyochelin synthase PchF	-6.743767	0.00284
Secretion system				
Q00513	xcpS	Type II secretion system protein F	-30.35565	0.001857
Q9I748	tssC1	Type VI secretion system sheath protein TssC1	-13.04946	0.0204823
Q00514	xcpT	Type II secretion system core protein G	-3.960423	0.00874
Transport and Efflux pumps				
P13794	oprF	Outer membrane porin F	-5.196342	0.004736
P32722	oprD	Porin D	10.82458	0.03093
P33641	bamD	Outer membrane protein assembly factor BamD	3.820441	0.0077372
Q51368	tonB	Protein TonB	-15.36099	0.00045
P11221	oprI	Major outer membrane lipoprotein	2.605767	0.000182

Proteins were classified according to their function. *Pseudomonas aeruginosa* database was downloaded from UniProt.

## Discussion

We have demonstrated that cell-free supernatants of the probiotic bacterium *E. coli* Nissle 1917 (Nissle) inhibit biofilm formation and disperse mature biofilm in *P. aeruginosa*. Nissle cell-free supernatants significantly reduced biofilm biovolume and dispersed biovolume that had developed for 24 h (Figure 2). Dispersion is an important phenomenon in biofilm and has been studied in detail previously in *P. aeruginosa* (Davies and Marques, 2009). In contrast, when testing the possible inhibitory effects of Nissle cell-free supernatants against the planktonic growth of *P. aeruginosa,* antibacterial effects were not observed (Figure 1). The lowest concentration of Nissle supernatant that significantly inhibited *P. aeruginosa* biofilm formation was 25% (*v*/*v*; Figure 3).

Further investigation of the effect of Nissle cell-free supernatant on *P. aeruginosa* biofilms using confocal laser scanning microscopy showed a significantly lower eDNA signal *P. aeruginosa* biofilms following exposure to Nissle supernatant during biofilm development (Figure 4), and a similar reduction in the eDNA associated fluorescence following the exposure of developed biofilms (Figure 5). While the mechanism(s) responsible for this are currently unclear, the involvement of nucleases is a possible explanation. The upregulation of DNAases was however not apparent in the proteomic data, and the lack of similar anti-biofilm activity in the other *E. coli* test strains suggests either that another mechanism is involved or that the nuclease profiles associated with Nissle are in some manner distinct.

It has recently been reported, using a similar methodology, that dead cells stained using TOTO-1 appear yellow due to the colocalization of red and green fluorophores, while eDNA appears green (Afshar et al., 2022). Lysed dead cells are a major source of eDNA within biofilms and hence may give a fluorescence signal at various steps in the intact but dead cell to the extracellular DNA pathway. In line with the current findings, a study investigating the effects of *E. coli* Nissle on *Clostridium perfringens* growth and virulence reported that Nissle inhibited biofilm formation, gas production and toxin production (α-toxin and NetB) in *C. perfringens*. However, the growth inhibition effect was not observed when *C. perfringens* was incubated with Nissle cell-free supernatants (Jiang et al., 2014). A study investigating the inhibitory effects of Nissle cell-free supernatant on *Salmonella* Typhimurium adhesion to porcine intestinal epithelial cells reported that Nissle cell-free supernatant inhibited *Salmonella* adhesion by down-regulating SiiE-mediated adhesion gene and, in agreement with the current study, Nissle cell-free supernatant did not inhibit the planktonic growth rate of the *Salmonella* (Schierack et al., 2011). These findings are also consistent with a recent investigation that reported anti-biofilm activity in spent cultures of several lactobacilli and bifidobacterial strains against two strains of *E. coli* (Abdelhamid et al., 2018). Inhibitory activities against *Vibrio* biofilms by *Lactobacillus* have also been reported (Kaur et al., 2018). In another report, supernatants derived from *Pseudomonas* sp. IV2006 SNIV2006 decreased biofilm formation and adhesion in *Flavobacterium* sp. II2003 without killing or suppressing growth (Doghri et al., 2020). Christofi et al. (2019) reported that *E. coli* can colonize the gut of healthy mice and protect them against intestinal colonization by *P. aeruginosa.*

To determine the type of component(s) responsible for the activity of Nissle cell-free supernatant against *P. aeruginosa* biofilm, enzymatic and heat treatments on the cell-free supernatant were performed. Proteinase K and heat treatment at 100°C removed the capacity of cell-free supernatant to inhibit biofilm formation (Figure 6), suggesting that the active substance includes heat labile proteinaceous factors. The pH of the cell-free supernatant was initially measured to exclude the potential of acidity-mediated anti-biofilm effects, and since the cell-free supernatant was pH neutral. Moreover, the cell-free supernatants were separated by size fractionation to determine the protein/s size involved in the anti-biofilm activity. The anti-biofilm activity was present in the 30 and 100 kDa fractions, which indicates the possible involvement of multiple proteins. No inhibition was observed in other fractions (Figure 7).

James et al. (1996) reported antibacterial and antibiofilm activity in proteins released by *Pseudoalteromonas tunicate* D2, and it has been additionally reported that proteinaceous exoproducts of the marine bacterium *Pseudoalteromonas* sp. 3J6 can inhibit the development of biofilms without affecting their planktonic growth (Klein et al., 2011; Rodrigues et al., 2015). Kim et al. (2009) demonstrated the capacity of *L. acidophilus-released* exopolysaccharide (r-EPS) to suppress EHEC biofilm formation. The r-EPS had no antibacterial effects on the planktonic form of EHEC but significantly inhibited biofilm production. The study reported that r-EPS influenced the initial adhesion and early autoaggregation steps in biofilm development. Petrova et al. (2016) described lectin-like compounds from in *L. rhamnosus* GG with anti-biofilm activity against *Salmonella* Typhimurium and uropathogenic *E. coli* biofilms. Ahn et al. (2018) reported that lipoteichoic acid is responsible for the anti-biofilm activity caused by *L. plantarum* supernatant against *Streptococcus mutans* biofilm.

We also assessed the potential protective effects of *E. coli* Nissle viable cells and the cell-free supernatants on a model of bacterial virulence based on waxworm moth larvae. *P. aeruginosa* viable cells were significantly lethal to the larvae but *E. coli* Nissle viable cells were markedly less so, and no significant toxic effects of cell-free supernatant of *E. coli* Nissle on the larvae were observed. This protection of pathogen-infected larvae may occur *via* direct pathogen suppression or effects involving the immune system of larvae. Direct pathogen suppression is probably most likely if protection is conferred when the probiotic is delivered to the larvae at the same time as the pathogen. In the current study, however, protective effects were not observed when Nissle viable cells and the cell-free supernatant and *P. aeruginosa* were injected concomitantly (Figure 8). Regarding host-dependent mechanisms (for example by activating the immune response of larvae by the probiotic), measurable protection of the larvae was not observed when Nissle viable cells were injected before the pathogen inoculation, but the cell-free supernatant was significantly protective against the lethal effects of *P. aeruginosa* only when administrated 24 h before the inoculation of the pathogen. This might be due to effects on the larval immune response that subsequently inhibited infection with *P. aeruginosa* (Figure 8). However, elucidating such mechanisms is beyond the scope of the current study. In a previous report, the ability of *Lactobacillus rhamnosus* GG, *Lactobacillus reuteri*, and *Streptococcus salivarius* K-12 to confer protection against the periodontal pathogens *Fusobacterium nucleatum*, *Porphyromonas gingivalis*, and *Aggregatibacter actinomycetemcomintans*, *G. mellonella* was assessed. Prophylactic exposure to candidate probiotics conferred protection (Moman et al., 2020). A separate study was conducted by a diffrent research group to explore the effects of the probiotic *Lactobacillus acidophilus* ATCC 4356 on biofilm development and *C. albicans* infection. Here, both *L. acidophilus* cells and filtrates inhibited biofilm formation by *C. albicans* and protected *G. mellonella* from the lethal effects of *C. albicans*, with an associated decreased microbial burden recorded in the larvae (Vilela et al., 2015).

In terms of other studies of protection by *E. coli* Nissle, it is reported that pretreatment with *E. coli* Nissle significantly inhibited the cellular invasion of *S. pullorum* in a chicken fibroblast model (Sun et al., 2022) and that supplementation with Nissle reduced *Campylobacter jejuni* colonization and enhanced the immune responses in infected chickens through the activation of the Th1, Th2, and Th17 pathways (Helmy et al., 2022). The protective effects of Nissle on human colonic cells infected with *Campylobacter jejuni* have also been investigated. Helmy et al. (2017) reported that pretreatment of HT-29 with Nissle conferred significant protection against invasion and intracellular survival of *C. jejuni* through increasing tight junction integrity and enhancing intestinal barrier function. In a similar study on the effects of Nissle on human colonic cells against *Campylobacter jejuni* infection, it was reported that the pretreatment of HT-29 cells with Nissle conferred significant protection against *C. jejuni* invasion by inducing anti-inflammatory cytokines and activating anti-apoptotic Akt signaling with associated protection against pro-inflammatory, and apoptotic responses induced by the campylobacter (Helmy et al., 2020).

In the current investigation, whole proteome analysis on *P. aeruginosa* biofilm cells after 24 h treatment with cell-free supernatant was done to gain an understanding of the potential mechanisms (Table 1; Figure 9). The expression of motility proteins related to flagella and pili was generally downregulated by at least 1.5-fold after treatment. Flagella and pili are recognized to be important in bacterial motility, adhesion to a surface, and movement within a biofilm. Including swimming, swarming, and twitching (Overhage et al., 2007; Giraud et al., 2011; De la Fuente-Nunez et al., 2012). The adhesion of bacterial cells to a surface is recognized to be one of the initial events in the creation of a biofilm. This process is aided by bacterial mobility in some situations (De la Fuente-Nunez et al., 2012). Some bacteria then begin to multiply and create extracellular polymeric compounds. Type IV pili are engaged in twitching motility, caused by pili extension and retraction (De la Fuente-Nunez et al., 2012). Twitching activity is involved in the cell-to-cell contacts required for the creation of microcolonies and the production of cell agglomerates characteristic of mature biofilms (Giraud et al., 2011). *P. aeruginosa* can swarm through viscous conditions in addition to swimming and twitching motility involving coordinated cell advancement across a semi-solid surface and is dependent on both flagella and type IV pili. Swarming aids in surface colonization and is critical in the establishment of early biofilms (Overhage et al., 2007).

It has been previously reported that antimicrobial peptides could target one of the major quorum sensing systems, rhl, by downregulating the expression of rhlA and rhlB proteins. The rhamnosyltransferase subunits rhlA and rhlB, are both important enzymes in the biosynthesis of rhamnolipids, which are bacterial surfactants that influence *P. aeruginosa* swarming motility and biofilm formation (Lin et al., 2018). In keeping with these findings are observed decreases in the synthesis of key molecules involved in *P. aeruginosa* pathogenicity and biofilm architecture, such as pyoverdine and rhamnolipids (Skariyachan et al., 2018). In the current investigation proteomic data suggest that treatment with cell-free supernatant of Nissle reduced the expression of RNA polymerase sigma factor RpoS and alginate-related proteins, e.g., positive alginate biosynthesis regulatory protein algR, alginate biosynthesis sensor protein KinB. It has been previously shown that these proteins influence the production of extracellular alginate, exotoxin A, and biofilm development in *P. aeruginosa* (Suh et al., 1999). Furthermore, secretion systems in *P. aeruginosa* are recognized to play important roles in *P. aeruginosa* virulence (Sultan et al., 2021). Treatment of *P. aeruginosa* with Nissle cell-free supernatant in the current study resulted in a significant reduction in type II secretion system-related proteins, e.g., xcpS and xcpT according to proteomic analysis. The type II secretion system of *P. aeruginosa* is involved in the extracellular release of numerous toxins and hydrolytic enzymes such as exotoxin A, lipases, phospholipases C, alkaline phosphatase, and elastase (Durand et al., 2003). A reduction level in type VI secretion system sheath protein TssC1 was also observed. *P. aeruginosa* within biofilm uses this enzyme to resist antibiotics (Zhang et al., 2011). Exposure to Nissle cell-free supernatant also resulted in up-regulation of the outer membrane porin D porD, outer membrane protein assembly factor BamD, and the outer membrane lipoprotein oprI. It has been reported that the basic amino acid-specific OprD porin in *P. aeruginosa* mediates the uptake of the β-lactam antibiotic imipenem (Trias and Nikaido, 1990; Huang and Hancock, 1993). Moreover, in *P. aeruginosa*, BamD interacts with T5SS to release proteins such as LepB and LepA (Meuskens et al., 2019), and oprI reportedly interacts with the peptidoglycan layer in *P. aeruginosa* (Duchene et al., 1989). However, in contrast, we observed a significant reduction level in other efflux pumps related proteins, the outer membrane porin F oprF, and TonB protein. The oprF is mainly engaged in several processes of *P. aeruginosa* infection, such as adhesion to eukaryotic cells (Azghani et al., 2002), and is known to be involved in biofilm development in cystic fibrosis (Yoon et al., 2002). Loss of OprF in *P. aeruginosa* PA14 induces susceptibility to a broad spectrum of antimicrobials, such as carbapenem (ertapenem), cephalosporins (cefotaxime), aminoglycosides (levofloxacin), tetracyclines (tigecycline; Dötsch et al., 2009), and the fluoroquinolone ciprofloxacin (Breidenstein et al., 2008). TonB is an energy-transducing protein that links the cytoplasmic membrane (CM) energy to several outer membrane receptors needed for the import of ferrisiderophores and other compounds that facilitate bacterial infection (Braun, 1995). Taken together, findings improve our knowledge of the mechanisms involved in the inhibitory effects of factors elaborated by *E. coli* Nissle against *P. aeruginosa*.

## Conclusion

*Escherichia coli* Nissle 1917 cell-free supernatants inhibited biofilm formation and dispersed mature *Pseudomonas aeruginosa* biofilms without inhibiting bacterial growth, and reduced eDNA in developing and extant biofilms. Physicochemical characterization of the putative anti-biofilm compound indicates the involvement of proteinaceous factors. Proteomic analysis showed a significant reduction in the expression of biofilm-associated proteins in *P. aeruginosa* treated with the cell-free supernatant. The cell-free supernatant had a significant protective effect in a *G. mellonella*-based larval virulence assay when administrated 24 h before challenge with the *P. aeruginosa*.

## Data availability statement

The raw data supporting the conclusions of this article will be made available by the authors, without undue reservation.

## Author contributions

AM, CO’N, and RL: conceptualization. AA and MA: performed the antibacterial and antibiofilm assays. AA and CE-C: performed the confocal microscopy and the proteomic experiments analysis. AA: performed the *Galleria mellonella* pathogenicity assay. AM, CO’N, and RL: supervision. AM and AA: writing the original draft. AM, AA, CO’N, and RL: writing, reviewing, and editing. All authors contributed to the article and approved the submitted version.

## Funding

This work was supported by a PhD studentship from the Ministry of Education (Saudi Arabia). The funder had no role in study design, data collection and interpretation or the decision to submit the work for publication.

## Conflict of interest

The authors declare that the research was conducted in the absence of any commercial or financial relationships that could be construed as a potential conflict of interest.

## Publisher’s note

All claims expressed in this article are solely those of the authors and do not necessarily represent those of their affiliated organizations, or those of the publisher, the editors and the reviewers. Any product that may be evaluated in this article, or claim that may be made by its manufacturer, is not guaranteed or endorsed by the publisher.
