# Supplementary material for: Escherichia coli Nissle 1917 inhibits biofilm formation and mitigates virulence in Pseudomonas aeruginosa
Source: Front Microbiol. 2023 Mar 8;14:1108273. doi: 10.3389/fmicb.2023.1108273 (PMC10031955; doi:10.3389/fmicb.2023.1108273)
Supplement: Supplementary file 1 [file Data_Sheet_1.docx]

**Supplementary information**

**Supplementary information 1 (Table 1). List of bacterial isolates that were tested in this study.**

| **Bacteria identification** | **Identification code** |
| --- | --- |
| *Brachybacterium rhamnosum* H-6S | MBRG AA 1.1 |
| *Corynebacterium afermentans* CIP 103499 | MBRG AA 2.1 |
| *Corynebacterium appendicis* IMMIB R-3491 | MBRG AA 2.2 |
| *Corynebacterium casei* LMG S-19264 | MBRG AA 2.3 |
| *Corynebacterium glyciniphilum* AJ3170 | MBRG AA 2.4 |
| *Corynebacterium mucifaciens* DMMZ 2278 | MBRG AA 2.5 |
| *Corynebacterium tuberculostearicum* MedalleX | MBRG AA 2.6 |
| *Corynebacterium Ureicelerivorans* | MBRG AA 2.7 |
| *Corynebacterium suicordis* P81/02 | MBRG AA 2.8 |
| *Dermacoccus nishinomiyaensis* DSM 20448 | MBRG AA 3.1 |
| *Kocuria arsenatis* CM1E1 | MBRG AA 4.1 |
| *Kocuria assamensis* S9-65 | MBRG AA 4.2 |
| *Kocuria palustris* TAGA27 | MBRG AA 4.3 |
| *Kocuria rhizophila* TA68 | MBRG AA 4.4 |
| *Micrococcus aloeverae* AE-6 | MBRG AA 5.1 |
| *Staphylococcus aureus* S33 R | MBRG AA 6.1 |
| *Staphylococcus auricularis* WK811M | MBRG AA 6.2 |
| *Staphylococcus capitis* JCM 2420 | MBRG AA 6.3 |
| *Staphylococcus caprae* DSM 20608 | MBRG AA 6.4 |
| *Staphylococcus epidermidis* NBRC 100911 | MBRG AA 6.5 |
| *Staphylococcus hominis* DM122 | MBRG AA 6.6 |
| *Staphylococcus hominis* GTC 1228 | MBRG AA 6.7 |
| *Staphylococcus auricularis* WK 811M | MBRG AA 6.8 |
| *Staphylococcus warneri* AW 25 | MBRG AA 6.9 |

MBRG, Manchester Biofilm Research Group.

**Supplementary information 2. Other *E. coli* cell-free supernatants did not inhibit biofilm formation of *P. aeruginosa***

The biofilm inhibitory effects of different *E. coli* supernatants were tested using crystal violet biofilm assay. Filtered supernatants were co-incubated together with *P. aeruginosa* in a Calgary biofilm device and incubated for 24 h. Biofilm produced by *P. aeruginosa* strain was significantly inhibited by Nissle cell-free supernatant with no inhibition observed by other *E. coli* strains which indicates the specificity of *E. coli* Nissle extract as anti-biofilm against *P. aeruginosa* compared to the supernatants of other *E. coli* strains (Fig S2).

**Figure S2:** The effects of supernatants of different *E. coli* strains on biofilm formation of *P. aeruginosa* (PA). The biofilm formation of *P. aeruginosa* was significantly inhibited only by *E. coli* Nissle cell-free supernatant. The white bar represents the control, and the hatched bars represent the pathogen treated with different *E. coli* supernatants. Data are presented as the mean ± SEM., n=3. * Indicates statistically significant difference (p<0.05) in biofilm formation.

**Supplementary information 3. *E. coli* Nissle cell-free supernatant did not reduce colony counts of *P. aeruginosa* biofilm viable cells**

After challenging mature *P. aeruginosa* biofilm with Nissle cell-free supernatant, both treated and untreated *P. aeruginosa* biofilms formed on pegs of Calgary device were detached and serially diluted in PBS then aliquots of 30 µl were plated in Luria agar and incubated for 24 h at 37°C. There was no significant difference in viable cells of *P. aeruginosa* biofilm when treated with Nissle cell-free supernatant compared to the control (Fig S3).

**
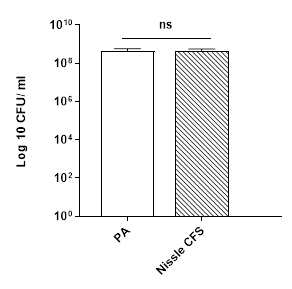
**

**Figure S3**: Cell viable count of *P. aeruginosa* (PA) biofilm in the presence and absence of Nissle cell-free supernatant (CFS) expressed as the mean log10 CFU/ml. The supernatant had no reduction effects on the viable cells within *P. aeruginosa* biofilm. Data are presented as the mean ± SEM., n=3.
